# *DISCO*: a general software tool for dislocation contrast factor calculations in diffraction analysis

**DOI:** 10.1107/S1600576726007053

**Published:** 2026-08-24

**Authors:** Marcelo Augusto Malagutti, Mirco D’Incau, Paolo Scardi

**Affiliations:** a Department of Civil, Environmental and Mechanical Engineering University of Trento Trento38123 Italy; DESY, Hamburg, Germany

**Keywords:** contrast factors, polycrystalline materials, single-crystal materials, transmission electron microscopy, computer programs

## Abstract

Dislocation contrast factors (CFs) play a central role in diffraction-based analyses of plastic deformation, linking the elastic strain fields generated by dislocations to diffraction line broadening. Despite their importance, the calculation of CFs remains a non-trivial task for low-symmetry crystal structures or arbitrary slip systems and is often limited to pre-tabulated solutions for selected lattice types. In this work we present *DISCO*, a software package for the real-time computation of dislocation contrast factors for any crystal structure and slip system. *DISCO* supports all space groups, from cubic to triclinic, and allows the calculation of CFs for individual crystallographic directions, as well as orientation-averaged CFs suitable for randomly oriented powder and polycrystalline diffraction analyses. The design principles, computational workflow and input/output structure of *DISCO* are described in detail, and its capabilities are illustrated through application examples on (cubic) SrTiO_3_, (hexagonal) 4H-SiC and (monoclinic) β-Ga_2_O_3_.

## Abstract 2

*DISCO* is a program capable of calculating the X-ray contrast factor for single- and polycrystalline materials in real time.

## 1. Introduction

The study of plastic deformation in materials spans a wide range of both fundamental and applied research fields, from geology to metallurgy and materials engineering. In this context, diffraction techniques have proven to be indispensable tools for investigating one of the most important microstructural defects governing plastic behaviour, namely dislocations.

Even before their existence was demonstrated and accepted as a physical mechanism of plastic deformation (Volterra, 1905 ▸, 1907 ▸; Orowan, 1934 ▸; Polanyi, 1934 ▸; Taylor, 1934*a* ▸,*b* ▸) it had been experimentally observed that cold work and the associated strain hardening led to a measurable broadening of diffraction lines. This broadening reflects the presence of long-range elastic strain fields and lattice disorder introduced by deformation. Once the key role of line defects in plasticity phenomena was recognized, theoretical descriptions of diffraction peak broadening were progressively refined, leading to models capable of providing quantitative estimates of dislocation density, character (edge, screw or mixed), and degree of interaction or spatial correlation.

A major step forward was achieved with the work of Wilkens, who established a theoretical framework allowing a direct comparison between theory and the entire diffraction line profile, yielding physically meaningful parameters related to dislocations (Wilkens, 1969*a* ▸,*b* ▸, 1987 ▸; Warren, 1990 ▸). In more recent years, the Krivoglaz–Wilkens formalism has been fully integrated into modern full-pattern analysis methods for diffraction data from powders and polycrystalline materials (Scardi *et al.*, 2018 ▸, 2001 ▸; Leoni *et al.*, 2007 ▸; Scardi & Leoni, 2002 ▸). Within this framework, conventional Rietveld refinement can be complemented by the whole powder pattern modelling (WPPM) approach (Scardi & Leoni, 2002 ▸; Scardi *et al.*, 2018 ▸), which enables the inclusion of physically based models for the main sources of peak broadening and shape. These range from the size, shape and size distribution of diffraction domains to microstructural defects, with dislocations playing a primary role (Scardi *et al.*, 2025 ▸; Rebuffi *et al.*, 2016 ▸; Leoni *et al.*, 2007 ▸).

In all theoretical expressions and models describing the effect of dislocation strain fields on the shape and width of diffraction peaks, it is necessary to account for the combined influence of two key factors: (i) the geometric relationship between the diffraction vector and the orientation of the line defects, and (ii) the elastic anisotropy of the medium, an intrinsic property of the investigated material. This information is contained in the elastic constants (either stiffness *C_ij_* or compliance *S_ij_* tensors) and in the specification of the slip systems, defined by the crystallographic slip planes and Burgers vector directions (Martinez-Garcia *et al.*, 2009 ▸; Ungár *et al.*, 1999 ▸).

Within this theoretical framework, these contributions are conveniently condensed into the contrast factor (CF), also referred to as the visibility factor in the context of trans­mission electron microscopy (TEM) (Meng *et al.*, 2021 ▸; Martinez-Garcia *et al.*, 2009 ▸; Ungár *et al.*, 1999 ▸). The CF quantifies the coupling between the dislocation strain field and a given diffraction vector. It can be computed numerically, and in some special cases also analytically. CFs may be evaluated for individual crystallographic directions, as required for single-crystal X-ray diffraction (XRD) studies or TEM observations, or averaged over symmetry-equivalent directions when dealing with powders or polycrystalline materials with randomly oriented grains and diffraction planes, under the assumption of uniformly populated slip systems.

In the latter case, it can be shown that the Fourier transform of the diffraction line profile associated with strain broadening due to dislocations can be written as (Warren, 1990 ▸; Wilkens, 1969*a* ▸,*b* ▸)
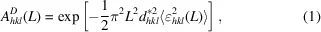
with
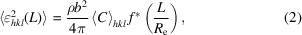
where *L* is the Fourier length, 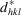 is the inverse of the lattice spacing for the *hkl* reflection, 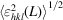 is the root-mean-square strain or microstrain, ρ is the dislocation density, *b* is the modulus of the Burgers vector, and 〈*C*〉_*hkl*_ is the powder average CF for the *hkl* reflection. 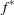
(*x*) is the Wilkens function defined by Wilkens (1969*a* ▸,*b* ▸).

In addition to its role within full-profile modelling approaches, the contrast factor also appears in several alternative formulations used for the analysis of powder diffraction data affected by dislocations. In the asymptotic limit of small Fourier lengths, expressions linking the Fourier coefficients of diffraction profiles to the dislocation density and contrast factor were derived for single crystals by Groma and co-workers (Groma, 1998 ▸; Groma *et al.*, 1988 ▸), and later extended to powder diffraction under the assumption of randomly oriented grains and uniformly populated slip systems (Groma & Székely, 2000 ▸). In this case, the resulting diffraction profiles are symmetric, and the effect of dislocations is fully captured by the real part of the Fourier coefficients, as in equation (1).

Related formulations are widely employed in Warren–Averbach-type analyses and in modified Williamson–Hall approaches for powders, where the contrast factor enters explicitly as a reflection-dependent scaling parameter (Ungár *et al.*, 1998 ▸, 2001 ▸; Scardi *et al.*, 2004 ▸; Ungár & Chung, 1999 ▸). In this case, the integral peak parameters such as the FWHM or the integral breadth (denoted β) are estimated, with crystallite size and microstrain parameters obtained by the relationship
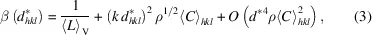
where *L*_V_ is the volume-weighted average crystallite size, and *k* is a constant that depends on the real cut-off radius, the Burgers vector modulus and numerical constants. Although these methods differ in their level of approximation and in the experimental observables they exploit, they all rely on an accurate determination of the contrast factor, which remains a key quantity for any diffraction-based analysis of dislocation microstructures.

Despite its central role in diffraction-based dislocation analysis, the calculation of CFs is still a non-trivial task, particularly for low-symmetry crystal systems or arbitrary slip systems. Existing software tools typically provide CFs only for a limited number of pre-calculated slip systems and are often restricted to cubic or hexagonal crystal symmetries (Borbély *et al.*, 2003 ▸).

In this article we present *DISCO*, a software package designed for the real-time calculation of dislocation CFs for any crystal structure or slip system. *DISCO* allows the computation of CFs along single crystallographic directions, as required for single-crystal diffraction or electron microscopy applications, as well as orientation-averaged CFs suitable for powder and polycrystalline diffraction analyses. In the latter case, the average contrast factor is obtained by averaging over all symmetry-equivalent slip-system directions of the crystal, assuming both a random grain orientation distribution and an equal population of all equivalent dislocation slip systems. The software supports any space group, from high-symmetry lattices down to the most general triclinic case, and does not rely on pre-tabulated solutions. Several examples are presented to illustrate the flexibility and applicability of the software to different materials systems and experimental configurations.

## 2. Theoretical basis

The contrast factor quantifies the coupling between the elastic strain field of a straight-line dislocation and a given diffraction vector **g**, where 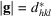. For a specified slip system and dislocation character φ, the CF corresponding to a single crystallographic direction can be written schematically as
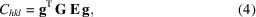
where **G** is the geometric matrix, which depends solely on the relative orientation between the diffraction vector, the slip plane, the Burgers vector and the dislocation line, while **E** is the elastic matrix, containing the contribution of the elastic anisotropy of the medium and derived from the elastic stiffness or compliance tensor (Martinez-Garcia *et al.*, 2009 ▸).

**Figure 1:**
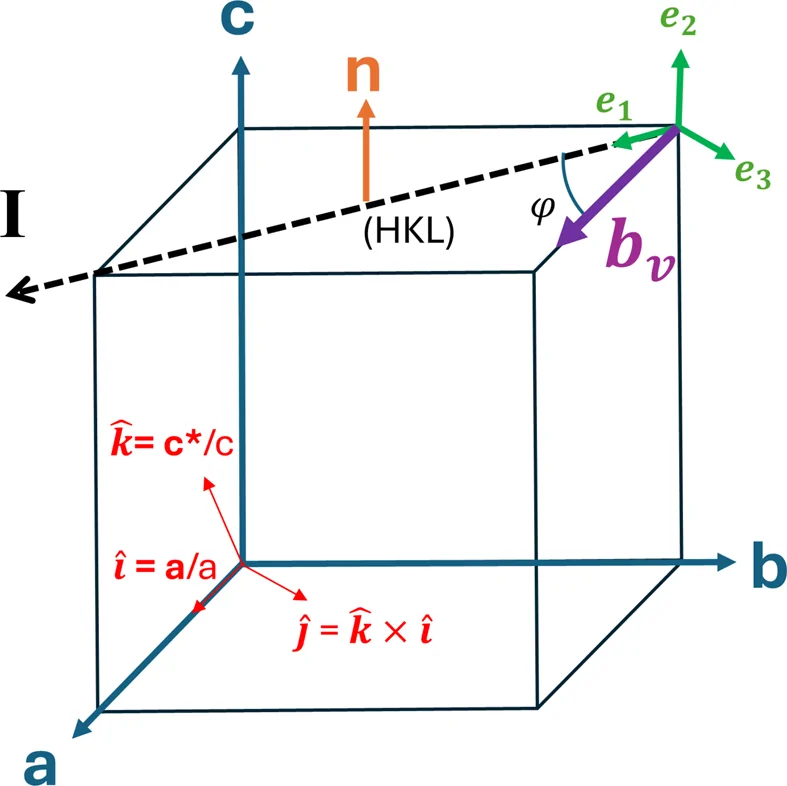
Schematic representation of a dislocation slip system, with the slip plane (*HKL*) having normal vector **n** and Burgers vector **b**_*v*_ in the 〈**uvw**〉 direction of the crystallite system. The (**a**, **b**, **c**) vectors correspond to the basis in crystallographic coordinates, whereas (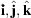) correspond to new orthonormal crystal frame coordinates constructed from (**a**, **b**, **c**). The slip-system frame is constructed from (**e**_1_, **e**_2_, **e**_3_), where **e**_1_ makes an angle φ with the Burgers direction and characterizes the slip system.

The geometric matrix **G** is constructed from the direction cosines τ_*i*_, which define the angles between the diffraction vector **g** and a local orthonormal reference frame associated with the slip system. To evaluate these quantities consistently, the crystallographic basis vectors (**a**, **b**, **c**) are conveniently transformed into an orthonormal Cartesian reference frame (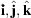) represented in Fig. 1 ▸. The slip-system reference frame, defined by the unit vectors (**e**_1_, **e**_2_, **e**_3_), is then constructed within this Cartesian basis following the procedure introduced by Martinez-Garcia *et al.* (2009 ▸):

(i) The unit vector **e**_2_ is chosen normal to the slip plane with Miller indices (*HKL*).

(ii) The unit vector **e**_3_ is taken along the dislocation line direction, lying within the slip plane and forming an angle φ with the Burgers vector 〈**uvw**〉, where φ = 0° corresponds to a pure screw dislocation and φ = 90° to a pure edge dislocation, and anything in between is considered to have a mixed dislocation character.

(iii) The third basis vector is obtained as **e**_1_ = **e**_2_ × **e**_3_, ensuring a right-handed orthonormal frame.

To evaluate the elastic contribution to the contrast factor, represented by the matrix **E**, the elastic tensor of the material is expressed in an orthonormal Cartesian reference frame 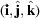. This tensor governs the relationship between the displacement field generated by a dislocation and its character (screw, edge or mixed) in an anisotropic elastic medium. The displacement field associated with a straight dislocation is obtained using the Stroh formalism (Stroh, 1958 ▸, 1962 ▸; Ting, 1996 ▸), which provides an exact solution for anisotropic elasticity and forms the basis for the computation of the elastic matrix **E** used in this work [see Martinez-Garcia *et al.* (2009 ▸) for details].

For powder or polycrystalline materials with randomly oriented grains and uniformly populated slip systems, the experimentally relevant quantity is the orientation-averaged contrast factor. This can be obtained by averaging the geometric contribution over all symmetry-equivalent configurations associated with the crystal point group and the selected slip systems, yielding
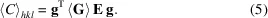


In *DISCO*, this averaging is performed explicitly at the level of the geometric matrix. Owing to the linearity of the construction, the use of an averaged geometric matrix **G** is formally equivalent to averaging the final CF values [like in equation (20) of Martinez-Garcia *et al.* (2009 ▸)], while offering a substantial reduction in computational cost. This strategy enables real-time CF calculations, even for low-symmetry crystal systems and complex slip-system configurations.

For powder diffraction, the dependence of the orientation-averaged contrast factor on the crystallographic direction is well established for all 15 Laue groups (Popa, 1998 ▸). This dependence can be expressed as a fourth-degree polynomial in the Miller indices, written in the general form
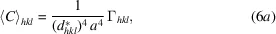

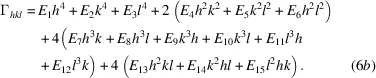
Here *a* is the reference lattice parameter used for metric normalization, such that the invariant polynomial Γ_*hkl*_ is rendered dimensionless and independent of the absolute unit-cell scale. Similar definitions are employed by Popa (1998 ▸), remaining consistent with the definitions of *E*_*i*_ in the literature and allowing its direct comparison. Depending on the Laue symmetry, the number of independent coefficients *E*_*i*_ is reduced, and the corresponding invariant forms are summarized in Appendix *A*[App appa].

The determination of these invariant coefficients is essential for powder diffraction analyses, as they provide a compact representation of the contrast-factor anisotropy that can be directly employed in whole powder pattern modelling (WPPM) and modified Williamson–Hall approaches (Scardi *et al.*, 2025 ▸, 2018 ▸, 2004 ▸). In *DISCO*, the coefficients *E*_*i*_ are obtained automatically by least-squares fitting of the calculated contrast factors over a set of diffraction planes.

## 3. Working principle

*DISCO* is a Python-based program that benefits greatly from the relative ease of that programming language for matrix multiplications. The input information is limited to known crystallographic and elastic parameters, while all remaining calculations for the orthonormal coordinate system of dis­location slip are determined automatically by the program. *DISCO* provides a detailed output, with contrast factor information on each equivalent slip system required for single-crystal XRD and TEM analysis.

### 3.1. Software architecture

Compared with other software tools available, such as *ANIZC* (Borbély *et al.*, 2003 ▸), or tabulated solutions reported by Ungár and co-workers (Dragomir & Ungár, 2002 ▸; Ungár *et al.*, 1999 ▸), the *DISCO* approach is fully general and does not rely on pre-defined slip-system tables. The calculation requires only the crystallographic definition of the slip system, given by the slip plane (*HKL*) and Burgers vector 〈**uvw**〉, together with the elastic properties of the material, and is applicable to all Laue symmetry groups.

**Figure 2:**
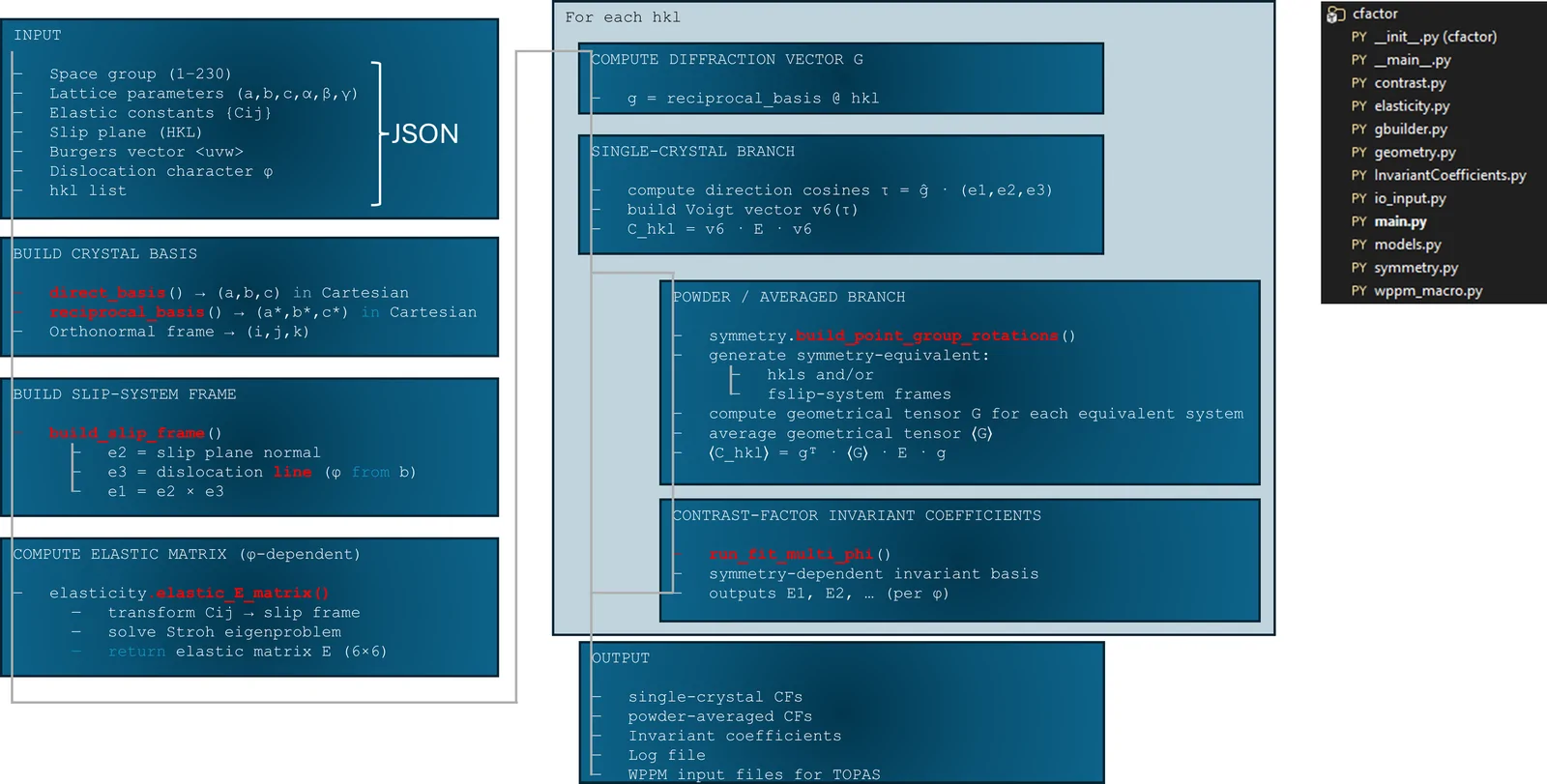
Algorithm of *DISCO* and the file structure.

The *DISCO* code is implemented using a modular architecture, designed to give a clear separation of the different conceptual and computational components involved in the calculation of dislocation contrast factors. This organization facilitates code maintenance, readability and future extensions, while closely reflecting the logical workflow of the algorithm illustrated in Fig. 2 ▸.

Input handling and data validation are performed by io_input.py, which parses the user-provided JSON input file and constructs validated internal data structures defined in models.py. The latter contains the core data classes (*e.g.* crystal structure, elastic properties, slip systems and dislocation character) that are shared consistently across all modules. Crystallographic transformations and coordinate handling are encapsulated in geometry.py, which provides routines to construct direct and reciprocal Cartesian bases from lattice parameters, as well as the local slip-system orthonormal frame (**e**_1_, **e**_2_, **e**_3_) used throughout the contrast-factor formalism.

Crystal symmetry operations are managed independently in symmetry.py, where the appropriate point-group rotations are generated from the space-group information. These symmetry operations are employed during the powder-averaging stage to generate symmetry-equivalent diffraction vectors and/or slip-system configurations, as depicted in the powder/averaged branch of the flow diagram in Fig. 2 ▸. The elastic contribution to the contrast factor is computed in elasticity.py, which transforms the elastic stiffness or compliance tensor into the slip-system reference frame and solves the corresponding elastic problem, returning the φ-dependent elastic matrix **E**.

The core calculation of single-crystal and powder-averaged contrast factors is implemented in contrast.py, which combines the geometric information derived from the diffrac­tion vector and slip-system frame with the elastic matrix to evaluate the contrast factor for each reflection. Finally, the op­tional determination of symmetry-invariant contrast-factor coefficients, intended for direct use in diffraction line-broadening models, is handled by InvariantCoefficients.py, with supporting routines in gbuilder.py to construct symmetry-appropriate invariant bases.

The outputs are generated within main.py itself for single-crystal and powder averages. Files required for WPPM in *TOPAS* (Coelho, 2018 ▸; Scardi *et al.*, 2018 ▸) are handled by wppm_macro.py, providing a ready-to-use snippet code.

### 3.2. Process

**Figure 3:**
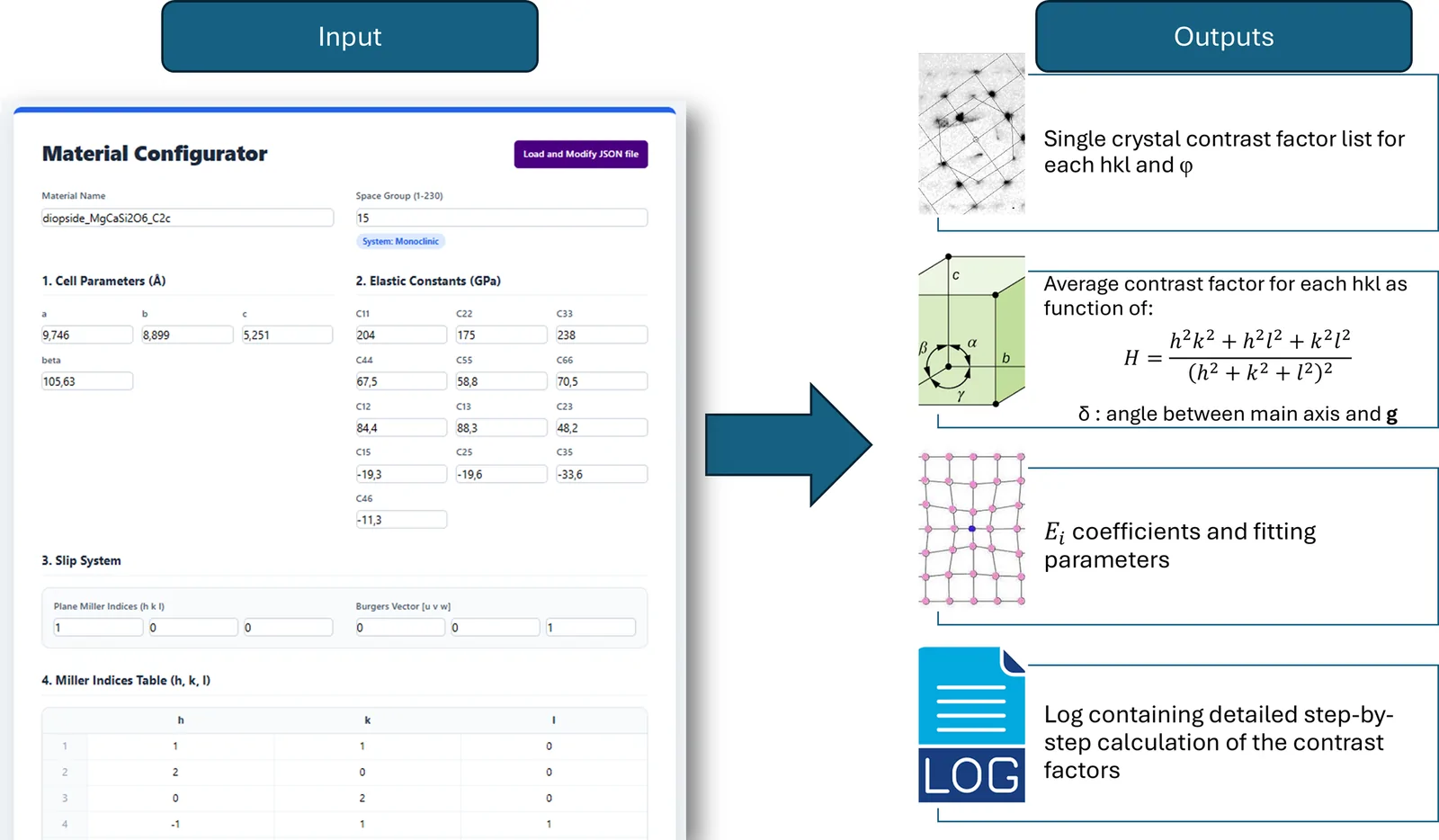
Example JSON input for the diopside monoclinic structure and the outputs generated by the code.

*DISCO* is operated through a command-line interface, taking a single JSON configuration file as input (Fig. 3 ▸). This input file can, however, be constructed in the graphical interface available at the program website (https://energymaterials.unitn.it/tools/software/disco.html). The input may define multiple slip systems and multiple dislocation character angles; *DISCO* processes all requested combinations in a single run and generates separate automatically named output files for each slip-system definition and φ value.

*DISCO* requires the following input information: crystal structure (lattice parameters and space group), elastic properties (stiffness or compliance tensor components), slip systems (slip-plane indices, Burgers vector directions and dislocation character angles) and diffraction vectors (list of *hkl* reflections of interest). All input fields are mandatory, and a series of examples are showcased in the supporting information.

Invariant symmetry coefficients *E*_*i*_, obtained by least-squares fitting of the calculated contrast factors, are written to comma-separated files (.csv) for each dislocation character angle. These coefficients can be directly employed in WPPM or modified Williamson–Hall analyses. For WPPM analysis in *TOPAS*, a .inp file is generated which can easily be copied and pasted into the *TOPAS* input file for Wilkens modelling of the microstrain. Invariant-coefficient files are generated automatically and may be ignored by the user if not required for a given application. Finally, a detailed log file (.run.log) is produced for each calculation, reporting the input parameters, symmetry operations, intermediate matrices and diagnostic information useful for validation and debugging. A summary of the most important information is also given when running the code directly from the website link.

*DISCO* supports batch execution in a single run. The input file may contain multiple slip systems and multiple dislocation character angles; *DISCO* evaluates all requested combinations and writes separate clearly named output files for each slip-system definition (plane/Burgers) and for each φ value. Similarly, users may provide arbitrarily long reflection lists and multiple materials/structures in the same input, enabling high-throughput contrast-factor generation for complex analyses or parameter sweeps. Invariant coefficients are computed automatically from the calculated contrast factors and saved alongside the contrast-factor tables; users may simply ignore these files if they are not required for a given workflow.

## 4. Examples and applications

### 4.1. Cubic SrTiO_3_

SrTiO_3_ is a prototypical perovskite oxide that exhibits pronounced elastic anisotropy and several crystallographically distinct slip systems (Hirel *et al.*, 2025 ▸; Gumbsch *et al.*, 2001 ▸). At room temperature, it crystallizes in the cubic *Pmm* structure with a lattice parameter of *a* = 3.905 Å (Howard *et al.*, 2004 ▸) and elastic constants of *C*_11_ = 316 GPa, *C*_12_ = 103 GPa and *C*_44_ = 122 GPa (Zhang *et al.*, 2022 ▸). The most commonly reported slip system in SrTiO_3_ is 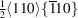, for which screw, edge and partial dislocations have all been experimentally observed (Brunner *et al.*, 2001 ▸; Hirel *et al.*, 2025 ▸). This case, in principle, cannot be solved with the current programs available in the literature for the powder average case (Borbély *et al.*, 2003 ▸). Single-crystal contrast factor calculations could be calculated in less than a second for all available software, and the calculation for the entire list of planes for this case study took on average ∼5 s using the website server.

**Figure 4:**
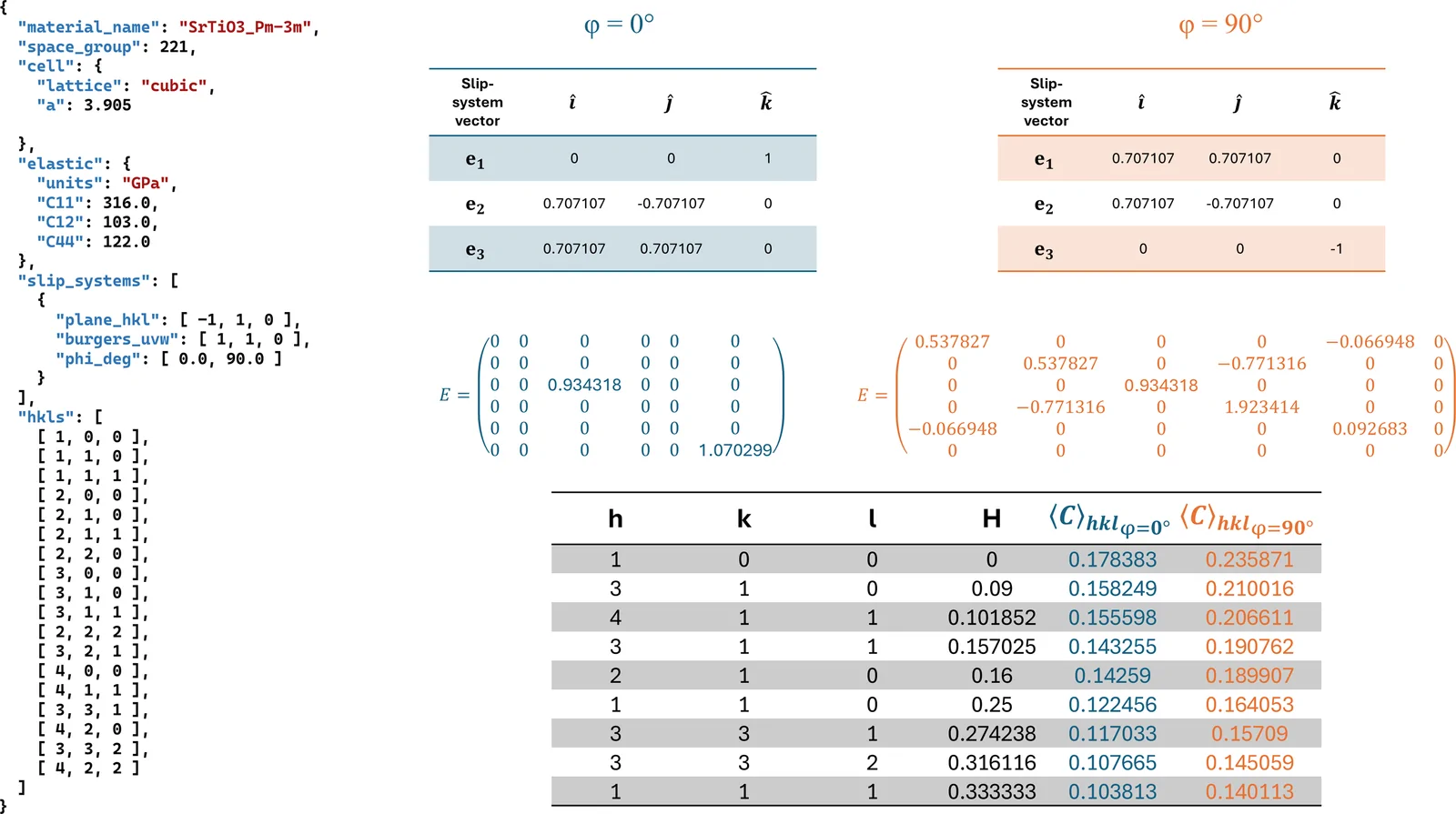
Input specification and calculated contrast factors for cubic SrTiO_3_ (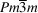) with slip system 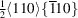, showing slip-system frames, elastic matrices in the slip reference frame and powder-averaged contrast factors for screw (φ = 0°) and edge (φ = 90°) dislocations.

The *DISCO* input file used for this case study, together with the resulting slip-system reference frames, elastic stiffness matrices expressed in the slip-system basis and powder-averaged contrast factors, are shown in Fig. 4 ▸. Calculations are performed for a selected list of *hkl* reflections and for both screw (φ = 0°) and edge (φ = 90°) dislocation characters. For each symmetry, only the essential lattice parameter information must be input, in this case ’a’: 3.905. The list of Miller indices is taken from the literature.

**Figure 5:**
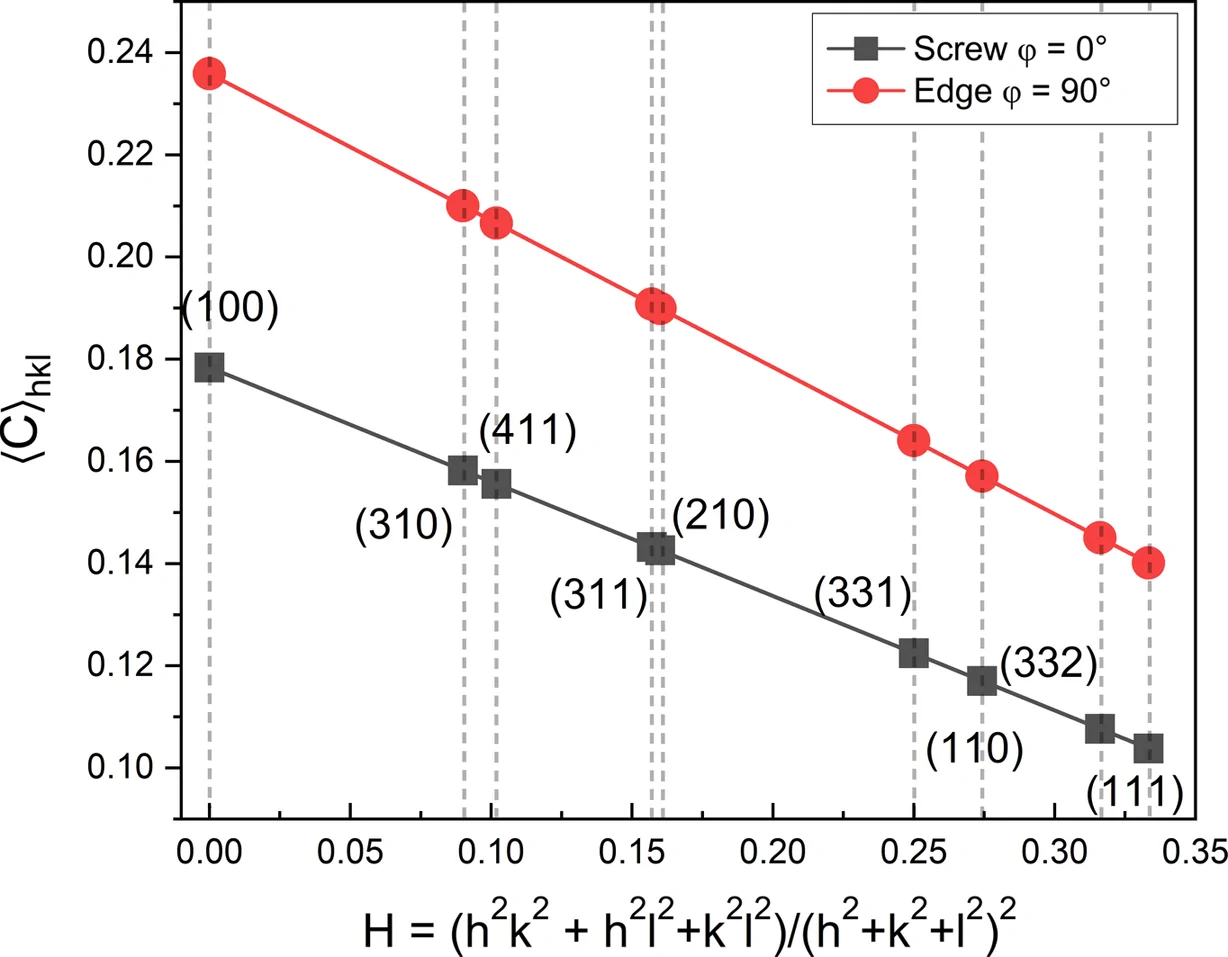
Powder-averaged contrast factors 〈*C*〉_*hkl*_ for cubic SrTiO_3_ plotted as a function of the cubic invariant. The black and red symbols correspond to the 〈*C*〉_*hkl*_ values for screw and edge dislocations, respectively. The straight lines correspond to linear fits for *A* and *B* as discussed in the text.

For cubic symmetry, the CFs can be conveniently represented in a linear form as
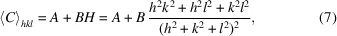
as shown in Fig. 5 ▸. Once the powder-averaged contrast factors are obtained, a least-squares fitting procedure based on the invariant expressions summarized in Appendix *A*9 is applied to determine the contrast-factor coefficients *E*_*i*_. In this form­ulation, *A* = *E*_1_ and *B* = 2(*E*_2_ − *E*_1_). Using the run_multi_phi() routine, *DISCO* automatically performs the fit independently for each dislocation character. For SrTiO_3_, the fitted coefficients are *A* = 0.1783 and *B* = −0.2237 for screw dislocations, and *A* = 0.2359 and *B* = −0.2872 for edge dislocations, with an agreement factor *R* ≃ 1 in both cases. The full input file is given in Supplementary Note 1 of the supporting information.

### 4.2. 4H-SiC

4H-SiC is a technologically important wide-bandgap semiconductor extensively used in high-power, high-frequency and high-temperature electronic devices (Chen *et al.*, 2024 ▸; Kimoto *et al.*, 2024 ▸; Poobalan *et al.*, 2024 ▸). It crystallizes in a hexagonal structure belonging to the space group *P*6_3_*mc* (No. 186), with pronounced elastic anisotropy and a complex dislocation landscape (Du *et al.*, 2023 ▸). At room temperature, the elastic stiffness constants reported from Brillouin scattering experiments are *C*_11_ ≃ 501 GPa, *C*_12_ ≃ 111 GPa, *C*_13_ ≃ 52 GPa, *C*_33_ ≃ 553 GPa and *C*_44_ ≃ 163 GPa, from which *C*_66_ ≃ 195 GPa can be derived (Kamitani *et al.*, 1997 ▸). These values are reported to be equivalent, within experimental uncertainty, to those measured for 6H-SiC and are commonly adopted for 4H-SiC as well.

Plastic deformation in 4H-SiC occurs through multiple crystallographically distinct slip systems. The most commonly observed mechanism is basal-plane slip on 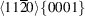, which is dominant under many deformation and growth conditions (Guo *et al.*, 2017 ▸). Prismatic slip on 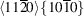 has also been reported and contributes to the overall dis­location population (Guo *et al.*, 2017 ▸). Dislocations in 4H-SiC can exhibit screw, edge and mixed character (Guo *et al.*, 2017 ▸); in particular, threading dislocations and basal-plane dislocations often possess mixed components. As a consequence, a realistic description of diffraction line broadening requires the ability to treat multiple dislocation characters, either individually or as weighted combinations.

**Figure 6:**
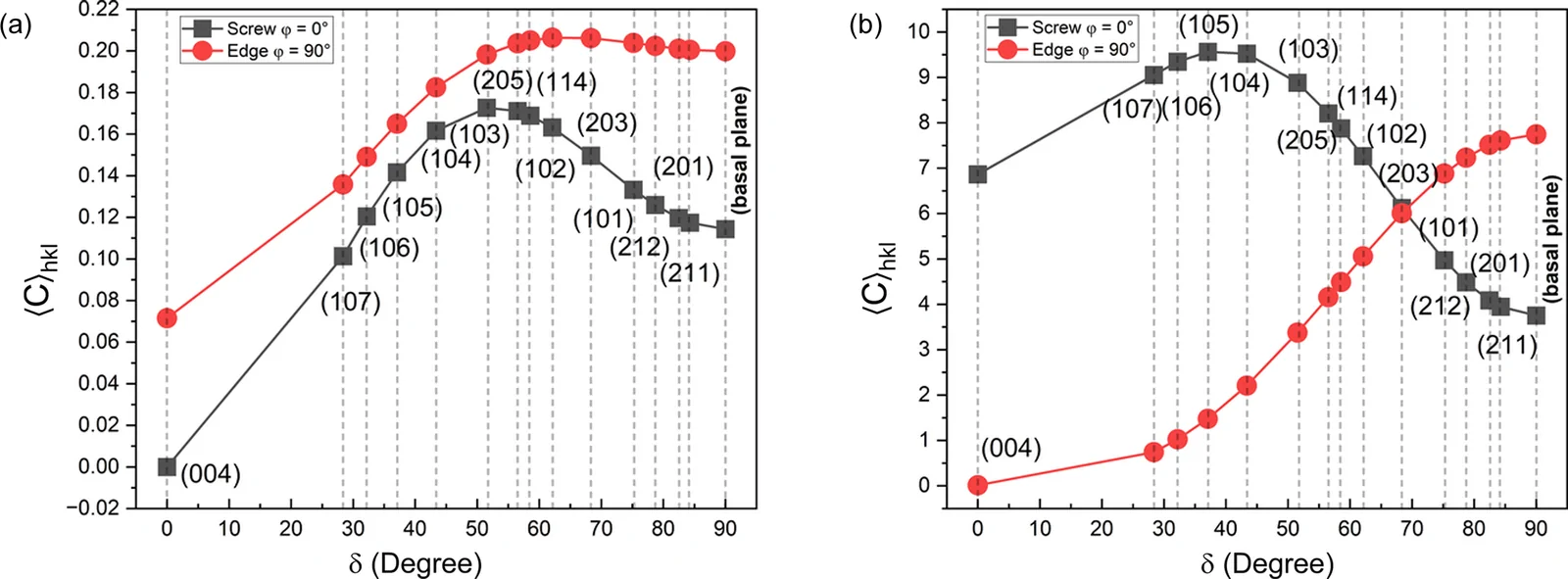
〈*C*〉_*hkl*_ versus δ, defined as the angle between the *c* axis and 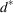, for screw (φ = 0°) and edge (φ = 90°) dislocations in hexagonal 4H-SiC. (*a*) The 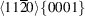 basal slip system. (*b*) The 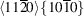 prismatic slip system. Individual reflections are marked by dashed vertical lines.

For hexagonal cases, it is interesting to plot the CF results as a function of the angle δ between the main axis *c* and the diffraction vector 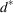,
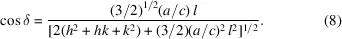


The results are given in Figs. 6 ▸(*a*) and 6 ▸(*b*) for the main and secondary slip systems of 4H-SiC, respectively, obtained in roughly 30 s. The contrast factor coefficients *E*_*i*_ are given in Table 1 ▸. The input files are given in Supplementary Note 2 of the supporting information. A pronounced difference in magnitude is observed between the two slip systems, with the secondary slip system exhibiting significantly larger contrast factors. This behaviour originates from the strong elastic anisotropy of 4H-SiC, in particular the high *C*_11_/*C*_13_ ratio, which enhances the coupling between the dislocation strain field and diffraction vectors with a 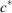 component. In contrast, basal slip is more efficiently accommodated by the axial stiffness *C*_33_, resulting in lower contrast factors. The smooth dependence of *C_hkl_* on δ confirms that these features are physically meaningful rather than numerical artefacts, demonstrating that *DISCO* can clearly distinguish different dislocation characters through a simple visualization.

### 4.3. β-Ga_2_O_3_

β-Ga_2_O_3_ is a wide-bandgap semiconductor (∼4.8–4.9 eV) crystallizing in a monoclinic structure, which has recently attracted significant attention for next-generation power electronics and deep-UV optoelectronics due to its high breakdown field and the availability of large single crystals (Higashiwaki *et al.*, 2012 ▸; Jamwal & Kiani, 2022 ▸; Zhang *et al.*, 2023 ▸). As device performance and reliability are strongly affected by crystallographic defects, a detailed understanding of dislocation structures and slip systems in β-Ga_2_O_3_ has become increasingly important.

**Figure 7:**
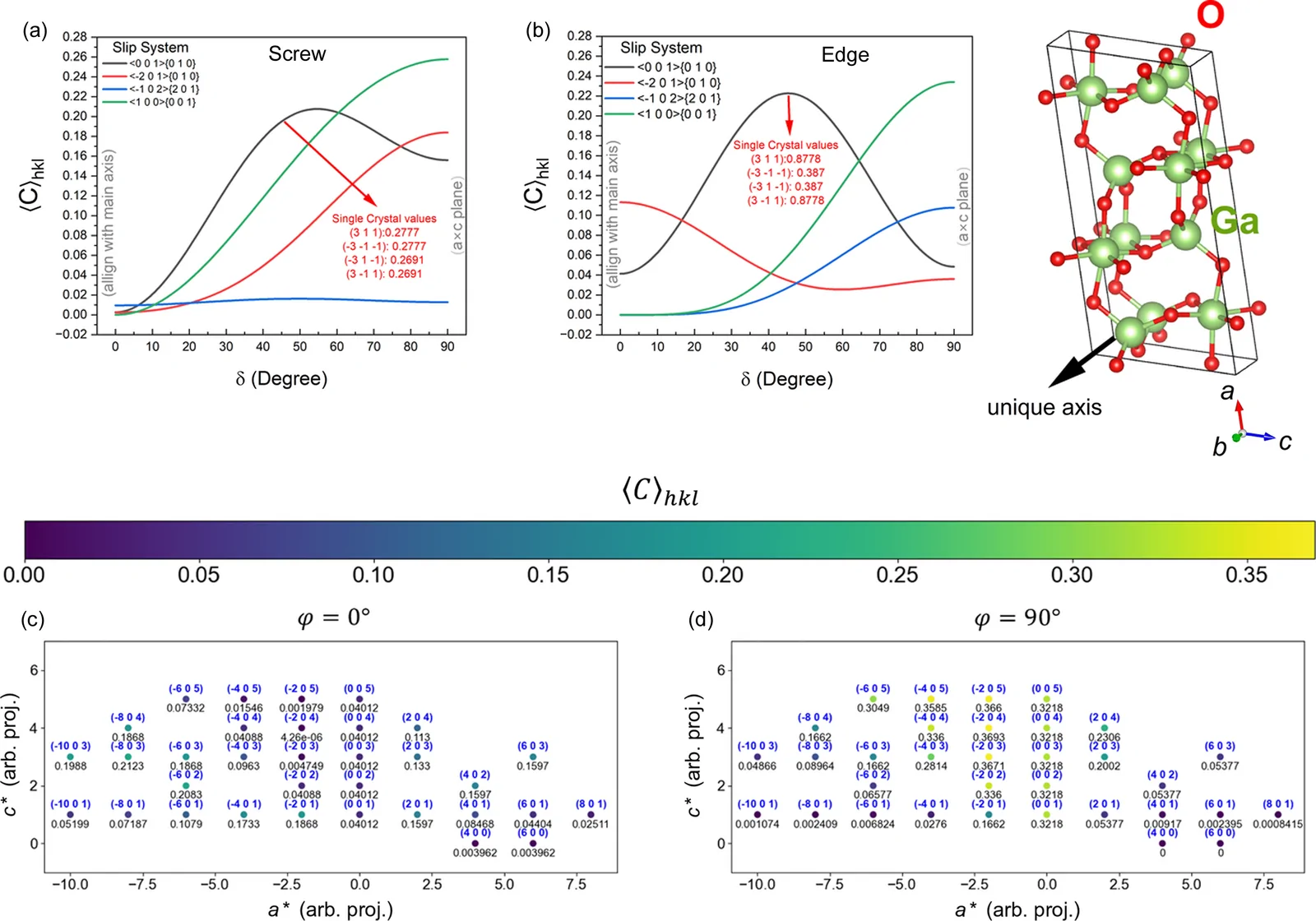
(*a*) Orientation dependence of the average contrast factor for screw dislocations in β-Ga_2_O_3_, shown as a function of the angle δ for several crystallographically distinct slip systems. (*b*) The same representation for edge dislocations, highlighting the strong anisotropy of 〈*C*〉_*hkl*_ and the corresponding single-crystal values. The crystal structure is shown on the right, indicating the monoclinic unit cell and the unique *b* axis. (*c*) and (*d*) Reciprocal-space maps of 〈*C*〉_*hkl*_ for φ = 0° (screw) and φ = 90° (edge), respectively, restricted to reflections with δ = 90°, *i.e.* scattering vectors lying in the a*c* plane. Only reflections associated with the 〈001〉{010} slip system are shown, with the colour scale representing the magnitude of the average contrast factor 〈*C*〉_*hkl*_.

The calculations were performed using the monoclinic β-Ga_2_O_3_ crystal structure with lattice parameters *a* = 12.2146 Å, *b* = 3.0374 Å, *c* = 5.8020 Å and β = 103.8748°, adopting the conventional unique-*b* setting (Yan *et al.*, 2024 ▸; Adachi *et al.*, 2018 ▸). The crystalline phase is represented in Fig. 7 ▸. The elastic constants *C_ij_* are *C*_11_ = 242.8 GPa, *C*_22_ = 343.8 GPa, *C*_33_ = 347.4 GPa, *C*_44_ = 47.8 GPa, *C*_55_ = 88.6 GPa, *C*_66_ = 104.0 GPa, *C*_12_ = 128.0 GPa, *C*_13_ = 160.0 GPa, *C*_23_ = 70.9 GPa, *C*_15_ = −1.62 GPa, *C*_25_ = 0.36 GPa, *C*_35_ = 0.97 GPa and *C*_46_ = 5.59 GPa (Yan *et al.*, 2024 ▸; Adachi *et al.*, 2018 ▸).

The smooth curves in Figs. 7 ▸(*a*) and 7 ▸(*b*) were obtained by evaluating the contrast factor along a continuous set of diffraction-vector directions spanning δ = 0°–90°, defined within the plane orthogonal to the monoclinic unique axis. Calculations took less than 5 s. At each angular position, the plotted values correspond to powder-averaged contrast factors, obtained by averaging over all symmetry-equivalent single-crystal contributions. Input files are given in Supplementary Note 3 of the supporting information. To elucidate the role of the diffraction geometry further, Figs. 7 ▸(*c*) and 7 ▸(*d*) report the reciprocal-space distributions of 〈*C*〉_*hkl*_ for δ = 90°, corresponding to diffraction vectors lying within the 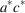 plane, for screw and edge characters. These maps reveal pronounced and highly anisotropic variations of 〈*C*〉_*hkl*_, particularly for the 〈001〉{010} slip system, with localized regions of enhanced contrast. Such behaviour arises from the combined effects of monoclinic symmetry and strongly anisotropic elastic constants in β-Ga_2_O_3_, which lead to a strong coupling between dislocation strain fields and specific reciprocal-space directions. The smooth and systematic evolution of the contrast factors across reciprocal space confirms that these features are intrinsic to the elastic response of the crystal rather than numerical artefacts.

## 5. Conclusions

*DISCO* provides a general, flexible and computationally efficient tool for the calculation of dislocation contrast factors in diffraction analysis. By combining a fully general symmetry treatment with an optimized computational strategy, the software enables real-time contrast factor calculations for arbitrary crystal structures and slip systems. *DISCO* is intended to facilitate the routine application of physically based dislocation models in diffraction-based microstructural analysis.

*DISCO* is freely available within GitHub at https://github.com/PaoloScardi/DISCO and on Zenodo at https://zenodo.org/records/18875171. An HTML graphical interface is also available from the authors’ website at https://energymaterials.unitn.it/tools/software/disco.html, allowing construction of the JSON files and running on the server. The present article should be cited when using *DISCO* in published work.

## Supplementary Material

Example files for DISCO. DOI: 10.1107/S1600576726007053/yr5167sup1.pdf

Zendo DOI including the source code: https://doi.org/10.5281/zenodo.18875171

## Figures and Tables

**Table 1:** Contrast factor coefficients for 4H-SiC See Appendix *A*8 for the parametric equations used for 〈*C*〉_*hkl*_.

	Screw (φ = 0°)			Edge (φ = 90°)		
Slip system	*E*_1_	*E*_2_	*E*_3_	*E*_1_	*E*_2_	*E*_3_
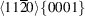	0.2031	0.0341	0	0.3552	0.0295	0.00062
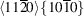	6.672	1.692	0.0599	12.591	0.1333	0.00099

## Appendix A Γ_*hkl*_ invariant expansions

This appendix reports the invariant-polynomial forms Γ used to represent the orientation-averaged contrast factors for powders. The expressions below follow the *TOPAS* macro style implemented in *DISCO* (*i.e.* written in terms of the Miller indices of the diffraction vector with respect to the reciprocal basis). For each space-group range (used as a practical proxy for the corresponding Laue class), we list the number of independent coefficients and the explicit Γ_*hkl*_ expression.

### A2. Monoclinic (space groups 3–15; Laue 2/*m*) – nine co­efficients

Two conventional unique-axis settings are handled:

Unique axis *b* (α = 90°, γ = 90°),

Unique axis *c* (α = 90°, β = 90°),

### A6. Trigonal (space groups 143–148; Laue 3) – five coefficients

If γ = 120°,

If γ = 60°,

### A7. Trigonal (space groups 149–167; Laue 3*m*) – four co­efficients

Trigonal  (space groups 150, 152, 154, 155, 156, 158, 160, 161, 164, 165, 166, 167):

If γ = 120°,

If γ = 60°,

Trigonal  (remaining space groups in 149–167):

If γ = 120°,

If γ = 60°,

### A8. Hexagonal (space groups 168–194; Laue 6/*m* and 6/*mmm*) – three coefficients

If γ = 120°,

If γ = 60°,
