# Supplementary material for: DISCO: a general software tool for dislocation contrast factor calculations in diffraction analysis
Source: J Appl Crystallogr. 2026 Aug 24;59(Pt 5):1720–9. doi: 10.1107/S1600576726007053 (PMC13636044; doi:10.1107/S1600576726007053)
Supplement: Supplementary file 1 [file j-59-01720-sup1.pdf]

JOURNAL OF APPLIED CRYSTALLOGRAPHY

Volume 59 (2026)

Supporting information for article:

DISCO: a general software tool for dislocation contrast factor calculations in diffraction analysis

Marcelo Augusto Malagutti, Mirco D'Incau and Paolo Scardi

Supplementary Note 1: SrTiO3


```
{
  "material_name": "SrTiO3_Pm-3m",
  "space_group": 221,
  "cell": {
    "lattice": "cubic",
    "a": 3.905
  },
  "elastic": {
    "units": "GPa",
    "C11": 316.0,
    "C12": 103.0,
    "C44": 122.0
  },
  "slip_systems": [
    {
      "plane_hkl": [
        -1,
        1,
        0
      ],
      "burgers_uvw": [
        1,
        1,
        0
      ],
      "phi_deg": [
        0.0,
        90.0
      ]
    }
  ],
  "hkls": [
    [
      1,
      0,
      0
    ],
    [
      1,
      1,
      0
    ],
    [
      1,
      1,
      1
    ],
    [
      2,
      0,
      0
    ],
    [
      2,
      1,
      0
```

```
  ],
  [
    2,
    1,
    1
  ],
  [
    2,
    2,
    0
  ],
  [
    3,
    0,
    0
  ],
  [
    3,
    1,
    0
  ],
  [
    3,
    1,
    1
  ],
  [
    2,
    2,
    2
  ],
  [
    3,
    2,
    1
  ],
  [
    4,
    0,
    0
  ],
  [
    4,
    1,
    1
  ],
  [
    3,
    3,
    1
  ],
  [
    4,
    2,
    0
  ],
  [
    3,
    3,
    2
```

```
      ],
      [
        4,
        2,
        2
      ]
    ]
}
```


Supplementary Note 2: 4H-SiC

1. $\langle 10\bar{1}0\rangle\{0001\}$


```
{
  "material_name": "4H-SiC_P63mc",
  "space_group": 186,
  "cell": {
    "lattice": "hexagonal",
    "a": 6.146,
    "b": 6.146,
    "c": 20.106,
    "alpha": 90.0,
    "beta": 90.0,
    "gamma": 120.0
  },
  "elastic": {
    "units": "GPa",
    "C11": 501.0,
    "C12": 111.0,
    "C13": 52.0,
    "C33": 553.0,
    "C44": 163.0,
    "C66": 195.0
  },
  "slip_systems": [
    {
      "plane_hkl": [
        0,
        0,
        0,
        1
      ],
      "burgers_uvw": [
        1,
        0,
        -1,
        0
      ],
      "phi_deg": [
        0.0,
        90.0
      ]
    }
  ],
  "hkls": [
```

```
[
  1,
  0,
  0
],
[
  1,
  0,
  1
],
[
  0,
  0,
  4
],
[
  1,
  0,
  2
],
[
  1,
  0,
  3
],
[
  1,
  0,
  4
],
[
  1,
  0,
  5
],
[
  1,
  1,
  0
],
[
  1,
  0,
  6
],
[
  2,
  0,
  0
],
[
  2,
  0,
  1
],
[
  1,
  1,
  4
],
```

```
    [
      2,
      0,
      2
    ],
    [
      1,
      0,
      7
    ],
    [
      0,
      0,
      8
    ],
    [
      2,
      0,
      3
    ],
    [
      2,
      0,
      5
    ],
    [
      2,
      0,
      6
    ],
    [
      2,
      1,
      1
    ],
    [
      2,
      1,
      2
    ]
  ]
}
```


2. $\langle 11\bar{2}0\rangle\{10\bar{1}0\}$


```
{
  "material_name": "4H-SiC_P63mc",
  "space_group": 186,
  "cell": {
    "lattice": "hexagonal",
    "a": 6.146,
    "b": 6.146,
    "c": 20.106,
    "alpha": 90.0,
    "beta": 90.0,
    "gamma": 120.0
  },
```

```
"elastic": {
  "units": "GPa",
  "C11": 501.0,
  "C12": 111.0,
  "C13": 52.0,
  "C33": 553.0,
  "C44": 163.0,
  "C66": 195.0
},
"slip_systems": [
  {
    "plane_hkl": [
      1,
      0,
      -1,
      0
    ],
    "burgers_uvw": [
      1,
      1,
      -2,
      0
    ],
    "phi_deg": [
      0.0,
      90.0
    ]
  }
],
"hkls": [
  [
    1,
    0,
    0
  ],
  [
    1,
    0,
    1
  ],
  [
    0,
    0,
    4
  ],
  [
    1,
    0,
    2
  ],
  [
    1,
    0,
    3
  ],
  [
    1,
    0,
    4
  ],
```

```
[
  1,
  0,
  5
],
[
  1,
  1,
  0
],
[
  1,
  0,
  6
],
[
  2,
  0,
  0
],
[
  2,
  0,
  1
],
[
  1,
  1,
  4
],
[
  2,
  0,
  2
],
[
  1,
  0,
  7
],
[
  0,
  0,
  8
],
[
  2,
  0,
  3
],
[
  2,
  0,
  5
],
[
  2,
  0,
  6
],
```

```
    [
      2,
      1,
      1
    ],
    [
      2,
      1,
      2
    ]
  ]
}
```


Supplementary Note 3: β-Ga_2O_3


```
{
  "material_name": "beta-Ga2O3_C2m_(001)_100",
  "space_group": 12,
  "cell": {
    "lattice": "monoclinic",
    "a": 12.2146,
    "b": 3.0374,
    "c": 5.802,
    "alpha": 90.0,
    "beta": 103.8748,
    "gamma": 90.0
  },
  "elastic": {
    "units": "GPa",
    "C11": 242.8,
    "C22": 343.8,
    "C33": 347.4,
    "C44": 47.8,
    "C55": 88.6,
    "C66": 104.0,
    "C12": 128.0,
    "C13": 160.0,
    "C23": 70.9,
    "C15": -1.62,
    "C25": 0.36,
    "C35": 0.97,
    "C46": 5.59
  },
  "slip_systems": [
    {
      "plane_hkl": [
        0,
        0,
        1
      ],
      "burgers_uvw": [
        1,
        0,
        0
      ],
      "phi_deg": [
```

```
      0.0,
      90.0
    ]
  }
],
"hkls": [
  [
    0,
    0,
    1
  ],
  [
    -2,
    0,
    1
  ],
  [
    2,
    0,
    1
  ],
  [
    4,
    0,
    0
  ],
  [
    -1,
    1,
    0
  ],
  [
    -4,
    0,
    1
  ],
  [
    0,
    0,
    2
  ],
  [
    -2,
    0,
    2
  ],
  [
    -1,
    1,
    1
  ],
  [
    1,
    1,
    1
  ],
  [
    -3,
    1,
    0
```

```
],
[
  4,
  0,
  1
],
[
  -3,
  1,
  1
],
[
  3,
  1,
  1
],
[
  -1,
  1,
  2
],
[
  -6,
  0,
  1
],
[
  6,
  0,
  0
],
[
  -3,
  1,
  2
],
[
  -2,
  0,
  3
],
[
  -5,
  1,
  1
],
[
  0,
  0,
  3
],
[
  -5,
  1,
  0
],
[
  4,
  0,
  2
```

```
    ],
    [
      -6,
      0,
      2
    ],
    [
      -4,
      0,
      3
    ],
    [
      6,
      0,
      1
    ],
    [
      5,
      1,
      1
    ],
    [
      2,
      0,
      3
    ],
    [
      -1,
      1,
      3
    ],
    [
      -3,
      1,
      3
    ],
    [
      -6,
      0,
      3
    ],
    [
      1,
      1,
      3
    ],
    [
      -8,
      0,
      1
    ],
    [
      0,
      2,
      0
    ],
    [
      -7,
      1,
      1
```

```
],
[
  -7,
  1,
  0
],
[
  -2,
  0,
  4
],
[
  -2,
  2,
  1
],
[
  5,
  1,
  2
],
[
  -7,
  1,
  2
],
[
  0,
  0,
  4
],
[
  -4,
  0,
  4
],
[
  2,
  2,
  1
],
[
  3,
  1,
  3
],
[
  8,
  0,
  1
],
[
  -4,
  2,
  0
],
[
  -4,
  2,
  1
```

```
],
[
  0,
  2,
  2
],
[
  -2,
  2,
  2
],
[
  -8,
  0,
  3
],
[
  2,
  0,
  4
],
[
  4,
  2,
  1
],
[
  2,
  2,
  2
],
[
  -4,
  2,
  2
],
[
  -5,
  1,
  4
],
[
  6,
  0,
  3
],
[
  -10,
  0,
  1
],
[
  5,
  1,
  3
],
[
  -6,
  2,
  1
```

```
  ],
  [
    -9,
    1,
    2
  ],
  [
    7,
    1,
    2
  ],
  [
    -9,
    1,
    0
  ],
  [
    -6,
    2,
    0
  ],
  [
    -6,
    2,
    2
  ],
  [
    -8,
    0,
    4
  ],
  [
    -2,
    0,
    5
  ],
  [
    -4,
    2,
    3
  ],
  [
    -4,
    0,
    5
  ],
  [
    -7,
    1,
    4
  ],
  [
    6,
    2,
    1
  ],
  [
    -9,
    1,
    3
```

```
    ],
    [
      9,
      1,
      1
    ],
    [
      -10,
      0,
      3
    ],
    [
      0,
      0,
      5
    ],
    [
      2,
      2,
      3
    ],
    [
      -6,
      0,
      5
    ]
  ]
}
```
